# Phenotypic Diversity and Abiotic Stress Tolerance Among *Vicia ervilia* (L.) Willd. Accessions

**DOI:** 10.3390/plants14071008

**Published:** 2025-03-24

**Authors:** Sofiya Petrova, Tsvetelina Stoilova, Valentin Velinov, Irina I. Vaseva, Lyudmila Simova-Stoilova

**Affiliations:** 1 Institute of Plant Genetic Resources “Konstantin Malkov” (IPGR), Agricultural Academy, Druzhba 2, 4122 Sadovo, Bulgaria; soniapetrova123@abv.bg (S.P.); tz_st@abv.bg (T.S.); 2 Institute of Plant Physiology and Genetics, Bulgarian Academy of Sciences, Acad. G. Bonchev Str. Bl 21, 1113 Sofia, Bulgaria; valentin.velinov82@gmail.com (V.V.); vaseva@bio21.bas.bg (I.I.V.)

**Keywords:** bitter vetch, phenotype variation, genetic breeding, drought, salinity

## Abstract

Bitter vetch (*Vicia ervilia* L. Willd.) is an ancient Mediterranean legume, well adapted to dry climates, that has recently gained attention for its potential in organic farming and as a suitable source of bioactive compounds. This study analyzed the agrobiological variability of 12 bitter vetch accessions from the IPGR-Sadovo genebank in two-year field trials. Yield-related traits were recorded, and grains were assessed for protein, sugar, starch, free amino acids, phenols, and antitrypsin content. Statistical analyses included variance, correlation, cluster, principal component, and path-coefficient methods. Significant variation was observed in plant branching, pod and grain numbers, and grain weight per plant. Grain yield correlated strongly with pod number (r = 0.910**), grains per pod (r = 0.867**) and per plant (r = 0.965**), and pod size. Positive direct effects on grain yield had the traits germination−50% flowering, number of seeds per plant, height to first pod, and harvest index. An indirect impact was found for the number of pods per plant, number of seeds per pod, and seed starch content. Accessions formed four main clusters. BGR6207, B9E0168, and C3000003 showed high yield potential. C3000001, C3000003, C3000007, and C3000006 exhibited early maturity. C3E0118, C3000007, and C3000003 seeds had lower amounts of phenols. BGR13526 presented lower protein and antitrypsin but higher carbohydrate and phenol levels. Tolerance to moderate osmotic stress (150 mM NaCl or 10% Polyethylene glycol 6000) varied. BGR3052, BGR13526, and A3BM0178 were found to be resistant to both stressors, while accessions C3000001 and C3000007 were identified as sensitive to both adversities. C3000006 was determined as sensitive to salinity but resistant to drought, and BGR3051and C3000003 were relatively sensitive to drought but resistant to salinity. Root elongation and thinning were observed in half of the accessions as adaptive responses to stress. These findings highlight some of the advantages of the evaluated bitter vetch accessions for breeding and reintroduction into sustainable agricultural practices.

## 1. Introduction

Legumes (family *Fabaceae*) are an indispensable source of complete food and feed, as well as of bioactive substances such as protease inhibitors, lectins, phytates, tannins, phenols, flavonoids, and others [1]. The genus *Vicia* (vetch) consists of about 160 annual and perennial species distributed in Europe, Asia, and America, in regions with a temperate climate [2,3]. It includes important species such as *Vicia ervilia* L. (bitter vetch), *Vicia narbonensis* (Narbonne vetch), *Vicia sativa* subsp. *sativa* (common vetch), and *Vicia villosa* subsp. *dasycarpa* (woolly vetch), which are grown for livestock feed in non-tropical dry areas. Vetch (*Vicia* spp.) is a unique multipurpose legume that can produce quality forage and grain. It can be incorporated into crop rotations as nitrogen fixing spp. while allowing for forage grazing, haying, and seed collection. It is also used for green manure and as a cover crop in rotation with cereals to improve their yield and soil quality [4,5,6].

Bitter vetch (*Vicia ervilia* (L.) Willd.) is a diploid (2n = 14), predominantly self-pollinating annual plant species of the genus *Vicia*, one of the earliest domesticated crops of the Fertile Crescent and one of the oldest grain legumes grown in Southern Europe, Western and Central Asia, and Northern Africa [7]. Bitter vetch was used in the past for human nutrition, and today it is mainly used for fodder and as a fortifying supplement [8,9]. Bitter vetch is used by sheep for grazing in early spring [10,11], while the grain is added in the rations of ruminants [11,12] and non-ruminants [13] as a cheap source of protein. Bitter vetch seeds have high nutritional value due to their high carbohydrate (66.2%) and protein (20.1%) content [14]. This species is regarded as an alternative crop in the context of the ongoing climate changes and the needs of organic farming and sustainable agriculture [15,16,17]. Recently, increased attention has been paid to the evaluation and improvement of bitter vetch germplasm to serve its conservation and to promote its potential for medicinal applications. Significant anti-inflammatory, analgesic, anti-ulcerogenic, antihyperglycemic, and antiviral activities have been found in the ethanol extract of bitter vetch seeds [8]. Seed extracts have been found to exert antioxidative effects and have been shown to inhibit tumor cell proliferation [9]. Several applications of bitter vetch seeds were described in a comprehensive phyto-pharmacological review [18]. For example, in traditional ethno-medicine, whole plants or decoctions are used for the treatment of digestive system pathologies and allergies, rheumatic pains, as palliatives, aphrodisiacs, or as a tonic for general weakness. Being a relatively drought-tolerant crop [4,17,19,20], bitter vetch is particularly suitable for small farming systems in arid marginal areas with neither chemical nor water input, as well as for organic agriculture and for the diversification of feed protein sources [15,16].

Little is known about the mechanisms of adaptation of *Vicia ervilia* L. to osmotic stress, especially drought and salinity [21]. Vegetative, reproductive, and grain-filling stages are critical for a legume’s grain quality and yield [19,22]. A linear relation has been established between grain yield in *Vicia* sp. and total rainfall, with critical periods—the beginning of flowering to seed formation [4]. Drought has a major impact on phenology and yield and less impact on grain quality parameters [19]. In semiarid conditions, low soil moisture at sowing causes delayed and irregular seedling emergence and establishment, which negatively affects yield [23]. The germination ability under osmotic stress in laboratory tests has been associated with water stress tolerance in field conditions [23]. Phenotypic variation was registered among common vetch genotypes in drought stress conditions [24] as well as under salt stress [25].

Local landraces are considered as a valuable material for the development of new varieties adapted for grain production, especially in arid highlands [26] and under organic agricultural systems in Southern Europe [15]. The presence of numerous accessions of local forms in seed banks worldwide enables the conservation of the genetic diversity in bitter vetch, which can be used to produce new varieties with better nutritional and yield characteristics. El Fatehi et al. [27] reported a wide range of variation in traits related to emergence, phenology, morphology, and yield among 19 bitter vetch ecotypes from northwestern Morocco. Abbasi et al. [28] assessed the genetic diversity of 126 samples from the Iranian genebank using agronomic–morphological traits and divided them into three main clusters. Russi et al. [15] characterized twenty-two Italian landraces by agro-morphological analyses as valuable material for breeding for organic farming and the use of seeds as a partial replacement of soybean in animal diets. Livanios et al. [29] assessed the phenotypic diversity among 49 bitter vetch Greek landraces covering 24 agro-morphological traits and classified them into eight distinct groups on the basis of traits related to the reproductive phase and grain yield, pointing out the importance of conservation and the reintroduction of local forms in cultivation and in breeding programs.

In Bulgaria, bitter vetch was grown in the past largely for feed for domestic animals. With the mechanization of agriculture, its cultivation has drastically diminished. Now, this crop occupies only relatively small areas in the southern parts of the country, where it is grown mainly for medicinal purposes. Having in view the potential of bitter vetch as an alternative crop to meet future climate challenges and the needs of organic farming and sustainable agriculture [15,16,17], our efforts were directed towards better characterization of local forms for their reintroduction in current agricultural practices. During numerous expeditions organized by the Institute of Plant Genetic Resources, Sadovo (IPGR) in the region of Southern Bulgaria, when examining small and large farms, no new local forms of *Vicia ervilia* L. were found. Therefore, this study covered only local forms from the *ex situ* collection of the IPGR, as well as repatriated samples from the genebanks of Gatesleben, Germany, and the USDA, USA. A comprehensive assessment of agro-morphological, biochemical, and yield-related traits as well as characterization of sensitivity to abiotic stress were conducted on a collection of twelve local bitter vetch accessions in order to evaluate their genetic variability and stress tolerance, with the goal of identifying potential donor lines for the breeding and reintroduction of bitter vetch in current sustainable agricultural practices.

## 2. Materials and Methods

### 2.1. Plant Material and Experimental Design

Twelve bitter vetch accessions were used in this study (Table 1), eleven of which were of Bulgarian origin. Five plant materials were repatriated from the USDA Germplasm Collection, USA. Five accessions were from the *ex situ* collection of the Bulgarian genebank, one was collected from a farmer during an expedition, and one Bulgarian local population was obtained from the IPK Gatersleben genebank, Germany. The experiments were conducted at the experimental field of IPGR-Sadovo on cinnamon forest soil, after a precursor of wheat during the years 2022 and 2023.

The experimental site is situated at longitude 24°57′ N, latitude 41°90′ E, and a 158 m altitude. Sowing was carried out manually at the optimal time (at the end of February), according to the recommendations for the cultivation of bitter vetch. Only the C3E0118 accession (autumn form) was sown at the beginning of November. The temperature and rainfall regime of the experimental site during the growth cycle are presented in Table 2. The area of Sadovo is characterized by a transitional continental climate with frequent and prolonged droughts. During the period 2022–2023 (coinciding with the plant life cycle), the mean temperature was 15.66 °C and the mean precipitation was 61.83 mm. The years 2022 and 2023 differed in meteorological conditions, especially rainfall, but this did not affect the normal development or yield of the studied crop.

The assessment of the economic traits of the accessions was performed according to the common vetch (*Vicia sativa* L.) descriptor list [30] and descriptor list from the IPK Gatersleben genebank, due to the absence of a descriptor list for bitter vetch. The field experiment was set as Randomized Complete Block Design (RCBD) with three replications. The grains were sown in 4 row plots, 1.5 m in length, with 0.50 m between rows and 0.25 m between plants. The distance between two plots was 100 cm. Accessions were grown under rain-fed conditions, without fertilizers, herbicides, or pesticides, and were hand-weeded when necessary.

### 2.2. Measurements of Traits

Several morphological and agronomical traits were measured and assessed during the two experimental years. During the growth period, the following parameters were evaluated: the start date of flowering, the days to 50% flowering and days to 80% maturing, and the duration of the whole growth cycle. Measurements were taken on 10 plants randomly selected from each plot. The estimated traits are listed in Table 3.

### 2.3. Biochemical Analyses of Grain Quality

Grains collected from the field trials (10 g per accession) were ground in a mill to fine powder. Seed flour (aliquots of 100 mg) was extracted with 1 mL of ice-cold 80% ethanol for 1 h on an ice bath with sonication and periodical vortexing. After centrifugation for 30 min at 14 500 rpm, 4 °C, soluble sugar, free amino acids, and phenols were analyzed in the supernatant by spectrophotometric methods modified for 96-well microplates, as described by López-Hidalgo et al. [31]. The pellets were additionally incubated in 30% perchloric acid for 1 h at 60 °C, and after centrifugation the second supernatant was used for starch content determination. Anthrone reagent was applied for the analysis of soluble sugar or hydrolyzed starch at 625 nm, using a standard curve for glucose [32]. Amino acid content was estimated with ninhydrin reagent at 520 nm with a calibration curve prepared by mixing equimolar standard solutions of L-proline and L-glycine [33]. Phenolic content was determined with Folin–Ciocalteu reagent at 720 nm using caffeic acid for the standard curve [34]. Optical density was registered using a Multiscan Spectrum (Thermo electron corporation, Waltham, MA, USA). The results were based on four independent extractions.

Trypsin inhibitory activity (TIA) was measured in the supernatant after extraction of 20 mg seed flour with 1 mL of 10 mM HCl. The method of Kakade et al. [35] was applied, consisting of preincubation of bovine trypsin with varying volumes of extract and estimation of the residual enzyme activity with benzoyl-DL-arginine-p-nitroanilide as a substrate at 410 nm. One unit of TIA was defined in the extract volume, giving 50% inhibition of bovine trypsin activity. For total soluble protein content, seed flour (50 mg) was extracted with 100 mM Tris–HCl buffer pH 7.8 containing 150 mM NaCl for 1 h in an ice bath with sonication. After centrifugation, protein content in the supernatant was determined by the method of Bradford [36] at 595 nm, with Coomassie Brilliant Blue G 250 (Sigma-Aldrich, St. Luis, MO, USA) and a standard curve for bovine serum albumin. The results for TIA and total soluble protein were based on three independent extractions.

### 2.4. Germination Tests in Control and Stressful Conditions

Bitter vetch seeds were surface-sterilized for 3 min in 12% sodium hypochloride, abundantly washed with distilled water, and then put to germinate in Petri dishes (9 cm diameter) on two layers of filter paper, 20 seeds per dish, with three independent replicas for each condition and 12 mL of distilled water (controls) or the same quantity of osmotically active solution (10% *w/v* polyethylene glycol (PEG) 6000 or 150 mM NaCl). Petri dishes were kept at constant temperature (25 °C) in the dark, and germination progress was followed daily. Seedling fresh weight (without cotyledons) and root and shoot length were registered after 120 h of germination. Data on germination are mean values from three independent experiments carried out in June 2024. The following germination parameters were registered: G%—percentage of germinated seeds (radicle ˃ 2 mm) from the total seed number; RST-G%—relative stress tolerance, expressed as the ratio GS/GC, where GS is germination percentage under stress and GC is the germination of control seeds; VI—seedling vigor index, expressed as G% × FW (mean fresh weight of the seedlings for each condition, without cotyledon); and RST-VI—relative stress tolerance expressed as the vigor index ratio under stress/control conditions.

### 2.5. Data Analysis

The collected data were processed using variation analysis [37] by determining a sample mean, the minimum and maximum value, the range of variation, and the coefficient of variation of each trait. The degree of variability of the traits, represented by the coefficient of variation (CV%), was indicated according to [38] as follows: up to 7.0%—very low; 7.1–12.0%—low; 12.1–20.0%—average; 20.1–40.0%—high; and over 40.1%—very high. One-way ANOVA (analysis of variance) was used to compare accessions against the control variety Rodopi or treatments against the control in germination tests. The mean values (M) were compared using three Least Significant Difference (LSD) values—0.1%, 0.05%, and 0.01%—regarding the mean value of the standard.

The relationship between the grain yield and its indices as well as the impact of some biological and biochemical traits on the grain yield were determined using correlation and path coefficient analyses. A total of 20 traits were included in these analyses (Table 4).

Correlation coefficients were established according to [37]. A weak correlation was designated when the correlation coefficient (r) was less than 0.33; at “r” values between 0.33 and 0.66, medium correlation was assigned, and a strong one was identified at “r” ranging between 0.66 and 0.99. To identify the similarity and proximity of the studied *Vicia ervilia* genotypes, hierarchical cluster analysis was applied [39]. The Euclidean distance among groups was used as a measure of genetic similarity. In order to overcome the difference in the dimensions of the studied traits, data were standardized beforehand. The results of the clustering are presented graphically by a dendrogram, showing the sequence of unification of the objects and the formed clusters. The studied genotypes are divided into groups, depending on the behavior of their characterizing traits relative to the average values [40]. The direct and indirect effects on the productivity of plants were expressed using path analysis [41]. The study also used factor analysis, showing the distances between genes in the genome controlling structural elements of the yield [42]. All experimental data were processed statistically using computer software SPSS for Windows Version 19.0 (IBM SPSS Statistics 19 Product Version: 19.0.0) [43].

## 3. Results

### 3.1. Morphological Evaluation

The morphological evaluation of the studied accessions is presented in Table 5 (stems and leaves) and Table 6 (flowers, pods, and grains). In all accessions, the lower petals lacked serrations and the pods were erect. Plants from all accessions exhibited strong basal branching and, except C3000001, a bushy erect growth habit. In most of the accessions, except BGR3051, BGR3052, and BGR6207, there was no anthocyanin in the stem. The flower size was medium, except in BGR13526, which has relatively small flowers, and C3000007, which was characterized with larger ones. The leaves were complex, delicate, pinnate, composed of oblong leaflets, and without tendrils at the end. The color of the leaves was of varying intensity, which grouped them in three distinct categories—green (eight accessions), yellow-green (three accessions), and light green (one accession—B9E0168). Five accessions had an elliptical leaf shape, six had a narrowly elliptical shape, and one accession (C3000007) had a broad elliptical leaf shape. The majority of the accessions had an even-pinnate leaf type, with only four accessions having odd-pinnate leaves (Table 5).

The accessions with one to three flowers in an inflorescence predominated (Table 6). Two accessions (B9E0168 and C3000007) had more than three flowers in an inflorescence. The studied samples were divided into three groups according to the standard color—pure cream (two accessions), cream with brown stripes (five accessions), light purple (three accessions), and pink (two accessions—BGR3051 and BGR13526). According to the color of the wings, the studied lines were divided into two groups—creamy (10 accessions) and pink (2 accessions).

The shape of the grains split the studied genotypes into two variants—rounded-angular (five accessions) and spherical (seven accessions). The ground color of the grains grouped the accessions into three distinct types—grains that were reddish-gray (six accessions), reddish-brown (five accessions), and reddish green brown (one accession—C3000003). Only two accessions (C3000006 and C3000001) had dot patterns on the grain testa. Overall, the studied accessions presented considerable morphological variability.

### 3.2. Phenotypic Variation in the Studied Parameters

The variation in twenty quantitative traits—economic, biological, and biochemical ones (described in Table 4)—was statistically assessed. Variability data were compared with the variety Rodopi (accession A3BM0178), as it is currently the only Bulgarian bitter vetch variety maintained for the market.

The results of the phenotypic variation in traits in the studied bitter vetch accessions during the two-year experimental period are presented in Table 7 (yield structure) and Table 8 (phenology and grain biochemical composition).

Plant height variation at maturity ranged from 24.7 cm to 64.2 cm, with an average height of 45.6 cm. The height of the first pod setting had amplitude of variation from 7.7 cm to 30.8 cm, with an average height of 21.9 cm. The highest plant height and height of the first pod setting was found in accession B9E0168, and the lowest in C3000001. The number of branches per plant varied from 0.7 to 4.0, being 1.9 on average. Significant variation was also found among accessions in the total number of pods per plant. The average number of productive pods for all accessions was 68.2, varying from 25.5 to 159.3. The number of grains per pod ranged from 2.1 to 4.0 on average for the two-year period, and the highest number (4.0) was in accession C3000003. The number of grains per plant had a value of 144.4 on average. The average size of the pod for the study period was 16.9 mm in length and 4.3 mm in width. The length of the pod varied from 12.5 to 22.4 mm, and accession C3000003 was characterized with the biggest pod size. The average grain weight per plant for all the studied accessions was 5.3 g. The average weight of 100 grains was 3.6 g. The average values of the harvest index ranged from 11.3% to 57.2%. The obtained harvest index, averaged over the two years, shows that 41.6% of the accessions exceed the control variety Rodopi (Table 7). Data on the yield structure showed that accession C3000003 (variety Borina, Bulgaria) was distinguished with the highest number of branches, number of pods per plant, number of grains per pod and per plant, weight of the grains per plant, and biggest pod size. Local populations BGR3051 and BGR3052 presented the lowest values of these traits as well as the lowest weight of 100 grains and lowest harvest index. The highest 100-seed weight was registered for accession C3000006, followed by C3000001, C3000003 and B9E0168. The highest harvest index was found in C3000001, followed by C3000002 and C3000006. The results obtained for the control variety Rodopi were close to the average values for most of the yield traits.

For estimating variation in phenology, the autumn form C3E0118 was excluded from the calculations as an outlier (as it has a very long growth cycle of 210 days compared to 112 days for the control variety Rodopi). The average duration of the germination–mass flowering sub-period was 64.5 days. The shortest duration (41 days) was registered for accessions C3000001 and C3000006, and the longest (83 days) for accession B9E0168 (Table 8). The shortest duration of flowering (first–last flower) (6 days) was in the Russian variety BGR13526, and the longest (14 days) was in the control accession A3BM0178, followed by C3000003 (13 days). The average duration of this sub-period was 10.3 days (Table 8). The whole growth cycle for the studied accessions varied from 83 to 162 days, with an average duration of 97.5 days, the differences between the accessions being 33 days. The variation in this parameter was within smaller limits in the early-ripening accessions, and the opposite tendency was documented for the late-ripening ones. This indicated preservation of the trait as a varietal particularity. In four forms of local origin (C3000001, C3000003, C3000007, and C3000006), a growth cycle with the minimum number of days (83 days) was recorded on average for the period, while the maximum number of days (162) was registered in B9E0168 (a local population from the village of Kondolovo).

The data on the biochemical traits of seed quality of the studied bitter vetch accessions, averaged for the two experimental years, are presented in Table 8. The average value of seed protein was 93.48 mg·g^−1^ FW, varying from 85.7 to 105.8 mg·g^−1^ FW. The highest protein content was measured in the local line B9E0168 and in the control variety Rodopi, while the lowest values were estimated for C3000007, BGR3052, BRG13526, and C3000003. Nine of the accessions had a lower total protein content compared to the control, with statistically significant differences. Regarding soluble sugars, it can be noted that the control accession had a lower content than a large number of accessions for this trait (B9E0168, BGR3051, BGR3052, BGR13526, C3000002, C3000003, and C3000007). The lowest soluble sugar content in seeds was registered in accession C3000006, while the highest was found in BGR13526. Seed starch content had an average value of 563.19 mg·g^−1^ FW and CV 7.7%. Negative differences compared to the control were proven only for three of the evaluated accessions (C3000002, C3000007, and C3E0118). A significantly higher seed phenolic content was detected in BGR13526. In C3E0118, C3000003, and C3000007, this parameter was lower than the one of the control variety Rodopi. The free amino acid content was in the range 0.030–0.077 mg·g^−1^ FW. Its lowest values were detected in the control variety, while accession C3000003 had the highest levels of free amino acids. The content of antitrypsins varied in the limits 1.220–2.550 U·mg^−1^ FW. Significantly higher values were registered in accessions BGR3051, BGR3052, C3000002, and C3E0118, while lower antitrypsin content was found in accessions BGR13526 and BGR6207. Data on seed biochemical composition showed that accession C3000003 (variety Borina) was distinguished, with relatively low protein but higher free amino acid content; it also had a low level of phenolics in the seeds. The accession BGR13526 (Russian variety Krasnodarskaia) presented lower protein and higher soluble sugar, starch, and phenolic content. The control variety Rodopi had high protein and lower soluble sugar levels in the seeds.

The variation summary data of the studied economic, biological, and biochemical traits in the studied accessions are presented in Figure S1 (Supplementary Material). The following traits expressed the highest variation among accessions: number of main branches (CV 60.5%), number of pods (CV 59%) and of grains per plant (CV 63.6%), and weight of grains per plant (CV 67.7%). Traits with an average degree of variability were as follows: plant height (CV 23.8%), height to first pod (CV 33.7%), time from germination to 50% flowering (CV 22.6%), duration of flowering (CV 24.3%), soluble sugars (CV 21.4%), phenols (CV 26.3%), and the content of amino acids in the grain (CV 29.4%). Seed starch and soluble protein content showed low variability (CV 7.7% and 6.8%, respectively).

### 3.3. Correlation Between the Studied Economic, Biological, and Biochemical Traits

The positive and negative correlations between the evaluated economic, biological, and biochemical traits in bitter vetch accessions are listed in Table 9. Traits related to yield structure displayed several strong correlations. Strong positive correlations were observed between plant height and height to the first pod and the number of pods per plant and the number of grains per pod and per plant, as well as the weight of the grains per plant. The number of grains per pod was in strong positive correlation with the number and weight of the grains per plant, the pod size, the weight of 100 seeds, and in medium-strength positive correlation with the harvest index. The number of grains per plant was in strong positive correlation with pod size. The length of the pods had a strong positive correlation with 100-seed weight and width of the pod, and a medium positive one with the harvest index. The harvest index depended positively on plant height but negatively on the height to the first pod. A medium-strength negative correlation was established between the height to the first pod and the pod size, as well as the 100-seed weight. The only trait without any significant correlations with other yield traits was the number of branches per plant.

Plant phenology traits had no significant correlations with the traits related to yield structure. Among plant cycle-related traits, a strong positive correlation existed between germination–50% flowering days and the total growth cycle duration. A medium-strength negative correlation was observed between the duration of flowering and seed soluble sugar content. Biochemical traits presented only medium-strength correlations—positive between seed amino acid content and antitrypsin activity and negative between phenolic and amino acid seed content, phenols and antitrypsins, and starch content and antitrypsins. Soluble sugar content in seeds was in a medium-strength positive correlation with the height to the first pod, starch content with pod width, and the number of grains per pod with the free amino acid content in seeds.

### 3.4. Hierarchical Cluster Analysis

**Figure 1:**
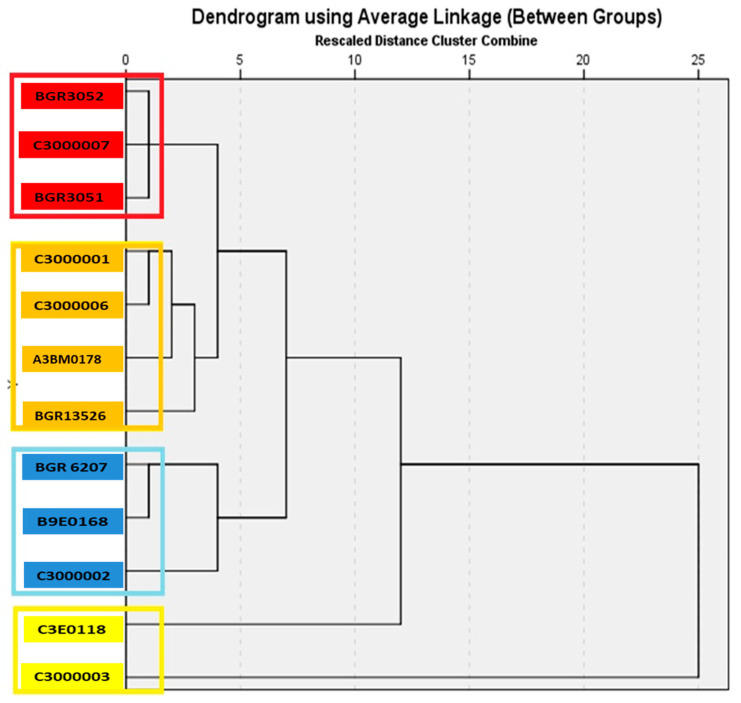
Dendogram for 12 bitter vetch (*Vicia ervilia* L.) accessions clustered by the average linkage method using economic, biological, and biochemical traits. The accessions on a yellow background are from the first cluster; those with a blue background are from the second cluster; those with an orange background are from the third cluster; and those with a red background are from the fourth cluster.

The assessment of the genetic proximity between bitter vetch accessions was carried out through the complex comparison of twenty traits (Table 4). According to the analyzed characteristics, the studied bitter vetch accessions were grouped into four main clusters (Figure 1).

The first cluster is the smallest and includes two accessions—C3000003 (variety Borina) and C3E0118 (local population), which are genetically the most distant from the other accessions included in this study. Borina is distinguished by high average values for the traits number of main branches, number of pods and grains from one plant, number of grains in one pod and weight of grains from one plant, length and width of the pod, weight of 100 grains, and early maturity, and has significantly higher amounts of amino acids and soluble sugars, and less phenols in the grain. C3E0118 has a greater grain weight per plant, more branches, a smaller amount of starch and phenols in the grain, but higher amino acid content and increased antitrypsin activity.

Three accessions (B9E0168, BGR6207, and C3000002) are included in the second cluster. They have high average values for the traits plant height, number of branches, number of grains in one pod, weight of grains per plant, longer pods, weight of 100 grains, early maturity, and content of sugars and amino acids in the grain.

The third cluster includes 33.33% of the evaluated accessions, including the control variety Rodopi, the Russian variety Krasnodarskaya (BGR13526), and two breeding lines. Among them, there are accessions with the highest average values of the studied traits. For example, breeding line C3000001 has a harvest index of 57.2% and a growth cycle of 83 days. Another breeding line from this group, C3000006, has a weight of 100 grains of 4.6 g and also a short growth cycle of 83 days. The Russian variety Krasnodarskaya has relatively high contents of sugars, starch, and phenols. This cluster also includes the control variety Rodopi (A3BM0178), distinguished by high soluble protein content.

Three accessions of Bulgarian origin (BGR3051, BGR3052, and C3000007) are included in the fourth cluster. They have above-average values for the traits plant height, first pod set height, soluble sugar content, and antitrypsin activity in the grain.

### 3.5. Principal Component Analysis (PCA)

PCA is a multivariate statistical technique for reducing a large number of correlated variables to a small number of principal factors. The values of the three components (PC1, PC2, and PC3) for each of the studied traits were calculated empirically (Table 10).

The analysis shows that the first component (PC1) explains 36.294% of the total variation, the second (PC2) 19.899%, and the third (PC3) 16.875%. The three factors together explain 73.068% of the total variation in the experiment, which is probably due to slightly lower levels of intrapopulation and higher levels of interpopulation diversity of the collection. This percentage illustrates the existence of complex interrelations between the studied traits. For example, the traits height to the first pod, number of pods per plant, number of grains per pod, number of grains per plant, weight of grains per plants, length of pod, width of pod, 100-grain weight, and harvest index are all related to the first component. The second component is in correlation with the traits germination–50% flowering, duration of flowering, growth cycle, starch, protein, phenols, and antitrypsin activity (Table 11). The remaining three traits—plant height, number of branches, and soluble sugars–are related to the third component.

**Figure 2:**
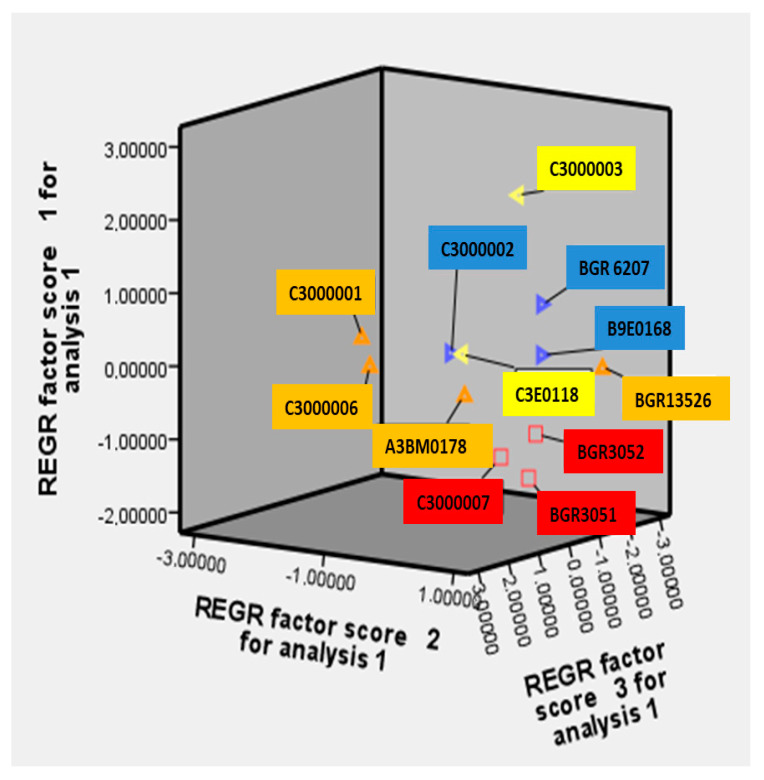
Distribution of the studied accessions in the factor plane. The accessions on a yellow background are from the first cluster; those on a blue background are from the second cluster; those on an orange background are from the third cluster; and those on a red background are from the fourth cluster.

The graphical representation of the studied traits illustrates their correlation within the frame of the studied twelve accessions (Figure S2). The results confirm the established correlation with high statistical significance and their distribution over the components PC1, PC2, and PC3. The distribution of the studied accessions in the coordinate system of PC1, PC2, and PC3 represents a grouping by similarity of the characteristics into one main group (Figure 2).

Several distinct “remote” accessions were positioned outside the group by this analytical approach. The accessions with numbers 9 (C3000001) and 11 (C3000006) fall into the negative quadrant of the first, second, and third factors, while the sample with number 8 (C3000003) falls in the negative quadrant of the third factor and in the positive part of the first and second factors (Figure 2 and Table S1). The Russian variety Krasnodarskaya with number 5 (BGR13526) is in the negative quadrant of the first and third factors and in the positive part of the second factor. The samples with numbers 9 (C3000001) and 11 (C3000006) are distinguished by short plants, a low setting of the first pod, a small number of branches, a large number of grains in one pod, a large number of grains from one plant, a large length and width of the pod, a high harvest index, short vegetation, few soluble sugars but more starch and amino acids in the grain, and increased antitrypsin activity. Sample 8 (C3000003) had the highest average values of traits, such as a high number of main branches and of pods and grains per plant, as well as a high number of grains in a pod, high weight of grains per plant, large length and width of the pod, high 100-grain weight, early maturity, higher amount of amino acids and soluble sugars, and less phenols in the grain. Sample 5 (BGR13526) has the highest content of sugars, starch, and phenols in the grain (Table S1). These accessions are also distinguished by a short growing season and are definitely of interest for hybridization according to individual characteristics and can be recommended as donors in the selection of bitter vetch.

### 3.6. Path Analysis of Productivity and Economic, Biological, and Biochemical Traits

With the help of path analysis, the direct and indirect effect of economic, biological, and biochemical traits on the grain productivity of a single plant can be traced. The following characteristics have the strongest direct influence on this trait: number of grains per plant (0.724); height to first pod (0.428); and harvest index (0.233) (Table 12). The number of pods per plant (0.698) and number of grains in one pod (0.566) have a strong indirect impact on the grain productivity of a single plant (Table 13).

The path analysis showed that the traits with higher positive direct effects on grain yield included germination–50% flowering, number of grains per plant, height to first pod, and harvest index, as well as amino acid, grain starch, and protein content. This implies that they can serve as indirect selection criteria to improve yield and the grain biochemical composition of bitter vetch.

### 3.7. Relative Stress Tolerance at Germination Stage

Information about the stress tolerance potential of the studied accessions could be additional selection criteria for including them in breeding programs. In preliminary experiments, we conducted a test with the control variety Rodopi, applying different concentrations of NaCl (75, 150, and 200 mM) and PEG 6000 (5, 10, and 15% *w*/*v*) to estimate the respective stress response and to select the most suitable stress parameters for comparative stress tolerance analyses among the accessions. The diminution in both germination rate and plant biomass was dependent on stress intensity (Figure S3). Under control conditions, the germination process of bitter vetch was almost completed after 48 h, with very fast radicle emergence in some of the accessions (C3000002, C3000003, C3E0118, and B9E0168). G% reached more than 50% in the first 24 h (Figure S4). The accessions BGR3051 and C3000006 had some germination issues, with relatively lower G% under control conditions. Diminished G% (Figures S5 and S6) and seedling vigor (Figures S7 and S8) was registered upon treatment with NaCl and PEG, with the most pronounced differences among accessions’ responses documented under moderate stress conditions. The vigor index was more severely affected by the applied stress than the germination rate. Moderate stress conditions were used for further comparison of the accessions in three independent experiments.

In Table 14 are presented the mean values for G%, VI, and RST for the studied bitter vetch accessions, measured after 120 h of germination in distilled water (controls), 10% PEG 6000, or 150 mM NaCl.

According to the calculated relative stress resistance regarding G% and VI, the following accessions were discerned: accessions sensitive to both types of stress (C3000001 and C3000007), resistant to both types of stress (BGR13526, BGR3052 and A3BM0178), and sensitive to salinity but resistant to drought (C3000006) and vice versa—relatively sensitive to drought but resistant to salinity (BGR3051 and C3000003).

The control variety Rodopi was relatively tolerant to drought stress. At moderate osmotic stress, significant elongation and thinning of the root is observed in half of the accessions as an adaptive reaction (Table 15). Accessions A3BM0178 and C3000003 presented root elongation under both PEG 6000 and NaCl stress. Accessions C3000001, C3000006, C3000007, and C3E0118 exhibited the same physiological reaction only when exposed to PEG treatment. Under salt stress conditions, accessions C3000001 and C3000007 had underdeveloped roots and arrested shoot development; therefore, they were not included in the measurements presented in Table 15. However, the observed root elongation under osmotic stress was not consistently related to genotype tolerance, as it was observed in some of the sensitive genotypes as well (accessions C3000001, C3000003, and C3000007). Also, some of the tolerant accessions (BGR13526 andBGR3052) did not show root elongation under osmotic stress.

## 4. Discussion

### 4.1. Phenotypic Variations Among the Studied Bitter Vetch Accessions

Evaluation of the phenotypic variations among bitter vetch local forms is a prerequisite for using some of them in breeding for desirable traits and reintroduction into agricultural practice. Our study established considerable variability among the studied traits in twelve bitter vetch accessions. The high genetic diversity in the studied collection is influenced by four highly variable traits (Table 7 and Table 8): number of main branches, number of pods and number of grains per plant, and weight of grains per plant. A high degree of variability was also presented for the following traits: height to the first pod, harvest index, time from germination to 50% flowering, and duration of flowering. Traits with an average degree of variability were the following: length of pod, 100-grain weight, growth cycle, and antitrypsin activity. The traits width of pod and starch content in the grain had a low degree of variability. Similar variability related to phenology, morphology, and yield was established by El Fatehi et al. [27]. Some of the evaluated traits are predominantly genetically determined, while others are highly influenced by environmental conditions. The accessions BGR3052, BGR6207, BGR13526, B9E0168, and C3000003 were distinguished by a high number of main branches per plant. This trait is genetically determined and is not significantly affected by fluctuations in the environmental conditions [44]. The correlation analysis (Table 9) did not reveal any relationship between the number of branches and the other studied traits. High-branching accessions could have different productivity—high (C300003), medium (BGR13526), and low (BGR3052).

Grain yield in bitter vetch is a quantitative trait that is influenced both by genetic factors and by changes in the environment. The number and the weight of the grains per plant, which are highly influenced by changes in the environmental conditions, are used as general traits for determining plant productive potential [4]. These traits showed the highest variability in the present study (Table 7). The harvest index depends on weather conditions, disease resistance, and agro-technical and stress factors [17]. The number of pods is also influenced by external conditions, pathogen attacks, the surface area of roots (which provide nutrients and water), and other morphological characteristics [45]. The experimental design of the present study involved comparative analyses of the tested accessions under the same environmental conditions. Therefore, the obtained results outline the accessions that exhibited high yield under local climatic conditions as potential candidates for utilization in selection programs.

According to the harvest index trait, five accessions, BGR6207, C3000002, C3000006, C3E0118, and C3000001, exceed the standard variety Rodopi. The average values of the harvest index in our study ranged from 11.3% to 57.2%. Similar intra-species variations in bitter vetch grain yield and harvest index were reported by other authors [28,29,46,47,48,49,50]. The presence of significant variation among genotypes for agro-morphological traits is indicative of the presence of a high degree of genetic variation useful for future selection, re-introduction, and/or utilization by breeding programs [29,51]. The formation of distinct clusters based on agro-morphological characteristics in local accessions of bitter vetch has also been established by other authors [28,52,53]. Saoub and Akash [4], by clustering of Jordanian vetch landraces, revealed that considerable variation for quantitative traits can exist between and within *Vicia sativa* and *Vicia ervilia* genotypes.

The duration of the entire growing period (germination–ripening) is determined both by hereditary characteristics and by environmental factors—temperature, humidity, light, etc. In our study, four accessions, C3000001, C3000003, C3000007, and C3000006, were distinguished by a short plant life cycle. Early-flowering accessions will be preferred in areas with temperate climates and low rainfall, while late-flowering forms will have higher yields in areas with high rainfall. Early flowering and pod maturity of accessions suggest that they could be used by farmers who require straw and grain for summer feeding. In addition, a shorter life cycle could favor plant reproduction in extreme climatic conditions. Two genotypes, BGR3051 and B9E0168, were distinguished by a long plant life cycle and harvest indices less than 30. Late-flowering accessions with harvest indices less than 0.30 would be suitable for spring grazing due to their long vegetative period [28]. Similar differences among bitter vetch accessions in the days from germination to the 50% flowering phase have also been reported in other studies [28,29,44,47]. The shortening or extension of this period depends on the sowing date. Later sowing usually corresponds to a shorter growing season due to the reduced time of the interphase periods [54,55]. The duration of the sub-period “germination–50% flowering” and the duration of the flowering period (first–last flower) depend on air temperature and humidity. Under cooler weather and optimal precipitation, these periods tend to be longer, while high temperatures and drought are factors that result in a relatively shortened plant life cycle. Thus, the duration of the growth cycle and the sub-periods germination–50% flowering and duration of flowering are inversely dependent on temperature and directly proportional to soil and air humidity. In our study, we established a strong positive correlation between the duration of the sub-period “germination–50% flowering” and the duration of the entire plant life cycle. However, no correlation was found between phenology traits and yield structure indices. Accessions with shorter or longer life cycles do not necessarily have an advantage in terms of productivity.

Seed biochemical composition added further variability among the studied bitter vetch accessions, discerning local forms with higher or lower protein content, higher or lower carbohydrate content, and a higher or lower level of phenols or antitrypsins, but these biochemical traits had no significant correlation with the studied yield related traits.

### 4.2. Yield Structure of the Studied Bitter Vetch Accessions

Indirect selection by characterizing yield-related elements can be more effective than direct selection by yield. Traditionally, correlation, regression, and path-coefficient analysis have been used to determine the relations between yield components and yield criteria in indirect selection [56,57,58]. The accessions BGR3051, BGR3052, BGR6207, BGR13526, B9E0168, C3000003, C3000007, and C3000002 were distinguished by taller plants. Plant height from a breeding point of view is an important trait for creating high-yielding varieties, with high grain quality, resistance to lodging, and suitability for mechanical harvesting [59]. The selection of bitter vetch is aimed at erect plants with reduced height in order to diminish or avoid damage from wind and rain, and these plants are therefore expected to be more resistant to lodging, as reported for other legume species [60,61]. The height of the first pod is of particular importance for mechanical harvesting [62]. According to our results, this trait is directly dependent on the total height of the plants. The number of pods on a plant correlated positively with the weight of the grains per plant, which defines it as an important trait for selection. The number of grains in a pod is a trait that has a certain role in the formation of the yield and is in a positive strong correlation with the weight of the grains per plant, which makes the trait very important for selection. The number of grains per plant determines the reproduction coefficient of the crop. It is related to the number of pods per plant and the number of grains in one pod. The correlation between the number and weight of grains per plant is particularly strong. Saoub and Akash [4], similarly to our results, established a strong correlation between grain yield and number of pods per plant and grain yield and number of grains per plant. The number of grains per plant is a very variable trait, which is strongly influenced by meteorological factors [17]. Very often, a larger number of grains in combination with a high 100-grain weight predetermine high productivity. In our study, such a potential was revealed in the old variety Borina (accession C3000003), which presented highest values of number of pods per plant, grains per pod, grains per plant, and size of pods, along with relatively high 100-seed weight. The size of the pod—length and width—is a trait that has a certain role in yield formation. The length and width of the pod are in a positive strong correlation with the number of grains per pod, which makes the trait very important for breeding. Factor analysis is a multivariate statistical technique for reducing a large number of correlated variables to a small number of principal factors [42]. The three factors together explain 73% of the total variation in our experiment, which is probably due to slightly lower levels of intrapopulation and higher levels of interpopulation diversity of the collection. Thus, our results for the traits related to the first principal component in the present study confirm previously published findings [17,28,29,63,64,65,66].

### 4.3. Stress Tolerance of the Studied Bitter Vetch Accessions

Screening for drought and/or salt tolerance at the germination stage has been suggested as a suitable approach to estimate large sets of germplasm for stress tolerance in general, as varieties tolerant to osmotic stress at germination were also considered to be drought-tolerant in field conditions [23]. Compared to other species of the genus *Vicia*, bitter vetch is less studied for stress tolerance at the germination stage [67]. Germinating *Vicia faba* L. has been predominantly tested in such analyses for establishing suitable grain priming protocols, highlighting the diverse response to priming among different varieties and species [67,68]. It has been reported that *Vicia villosa* Roth germinates well in a wide range of temperature (15–25 °C) and pH (5–10) variations, with a strong inhibition of germination at a pH below 4. This species exhibited salt tolerance but was relatively sensitive to osmotic stress [69]. Similar differential tolerance to these two osmotic stressors was reported by Perissé et al. [70]. In our study, we identified four distinct combinations in the response of *Vicia ervilia* L. accessions to moderate PEG osmotic stress and to salt stress—tolerance to both, sensitivity to both, tolerance to drought but sensitivity to salt, and tolerance to salt but sensitivity to drought stress. This divergent tolerance could be partially explained by the different nature of salt and drought stress, as salt stress adds ion toxicity to the osmotic effect [25]. Similar effects of osmotic stress on germination timing and percentage, as well as on seeding vigor, have been reported by other authors for bitter vetch [71] as well as for other legume species [23,72,73]. Generally, moderate osmotic stress exerts less negative effect on the germination rate but drastically reduces seedling vigor [73,74]. The mechanisms underlying the differential tolerance to salt and osmotic stress in some of the studied accessions and the correlation between stress tolerance at germination and at later plant growth stages (vegetative and reproductive) need further elucidation. However, the obtained information on the stress responses of the accessions under study could be a useful selection criterion for breeding purposes.

## 5. Conclusions

The results obtained from the analyses revealed a great diversity in important agro-morphological, economic, and biochemical characteristics, as well as in the drought and salt stress responses of the studied bitter vetch accessions, collected from the main areas of this geographic region that are historically known for the cultivation of this crop. The accessions BGR3051, BGR3052, BGR6207, BGR13526, B9E0168, C3000003, C3000007, and C3000002 exhibited a higher stem height compared to the control variety, without showing a tendency for lodging. Five of the accessions (BGR3051, BGR3052, BGR 6207, BGR13526, and B9E0168) had a significantly higher first pod setting than the control. Two accessions (BGR6207 and C3000003) had a large number of pods per plant, grains per pod, and grains per plant. Some of the accessions (BGR6207, BGR13526, B9E0168, C3000001, C3000002, C3000003, and C3000006) exhibited a significantly higher 100-grain weight compared to the control. Some accessions with a greater average weight of grains per plant for the study period stood out—BGR6207, C3000003, C3E0118, B9E0168, and C3000002. According to the harvest index averaged for the two years, five accessions (BGR6207, C3000002, C3000006, C3E0118, and C3000001) exceed the control variety Rodopi.

Accessions BGR6207, B9E0168, and C3000003 are characterized by a good yield potential, while the feature “earliness” was assigned to accessions C3000001, C3000003, C3000007, and C3000006. Accessions BGR3052, C3000006, C3000003, C3000001, C3000007, and C3000002 have demonstrated increased seed antitrypsin activity and reduced phenolic content. Accessions BGR3052, BGR13526, and A3BM0178 present a high tolerance to both drought and salt stress at the germination stage.

To develop varieties suitable for mechanized harvesting and that can serve as starting material for combinatory selection, the accessions BGR3051, BGR3052, BGR 6207, BGR13526, and B9E0168 could be recommended. Accessions BGR6207, C3000003, C3000002, and B9E0168 could be used as parental pairs in combinatory selection for high yield and productivity. The high frequency of highly significant variation among local forms for most of the studied traits highlights their excellent potential as a valuable bio resource for future breeding programs aimed at improving yield, its components, earliness, grain biochemical composition, and stress tolerance.

## Figures and Tables

**Table 1:** Passport information of the tested *Vicia ervilia* (L.) accessions.

Accession	Name	Biological Status	Origin	Source
A3BM0178	Rodopi	Variety	Bulgarian	IPGR genebank
BGR3051		Local population	Bulgarian	IPGR genebank
BGR3052		Local population	Bulgarian	IPGR genebank
BGR 6207		Local population	Bulgarian	IPGR genebank
BGR13526	Krasnodarskaia	Variety	Russian	IPGR genebank
C3000001	CPI 10385	Breeding line	Bulgarian	USDA, USA genebank
C3000002	SH B7-3-3-1	Breeding line	Bulgarian	USDA, USA genebank
C3000003	Borina	Variety	Bulgarian	USDA, USA genebank
C3000006	B92-198	Breeding line	Bulgarian	USDA, USA genebank
C3000007	B92-200	Breeding line	Bulgarian	USDA, USA genebank
C3E0118		Local population	Bulgarian	Ustrem village, Topolovgrad
B9E0168		Local population	Bulgarian	IPK Gatersleben genebank

**Table 2:** Meteorological data at the experimental region during plant life cycle.

	7 Feb–7 March	8 March–5 April	6 April– 4 May	5 May– 2 June	3 June– 1 July	2–31 July
Year 2022						
Minimum temp. (°C)	−0.73	3.98	5.39	10.14	15.51	15.53
Maximum temp. (°C)	9.10	11.36	18.27	25.47	27.79	31.32
Mean temp. (°C)	4.95	6.71	14.01	19.53	23.00	25.65
Rainfall (mm)	62.80	9.50	53.10	69.80	116.50	116.50
Year 2023						
Minimum temp. (°C)	−0.51	2.69	6.70	10.44	14.42	18.09
Maximum temp. (°C)	11.19	14.51	16.57	20.26	26.77	32.81
Mean temp. (°C)	6.33	9.55	12.54	16.33	22.07	27.20
Rainfall (mm)	17.00	44.00	31.20	78.80	80.20	62.50

**Table 3:** Morphological and agronomical traits studied in the accessions of bitter vetch.

Trait	Abbreviation	Classes
*Vegetative phase*		
Serration of lower leaves	SLL	1: serrate; 2: dentate; 3: entire
Stem anthocyanins	SA	1: absent; 2: present
Plant Branching	PB	1: strong basal; 2: only in the upper part
Presence of tendrils	PT	1: absent; 2: present
Presence of leaves	PL	1: absent; 2: present
Leaves color	LC	1: yellow green; 2: green; 3: light green
Leaves shape	LSh	1: narrow elliptic; 2: elliptic; 3: broad elliptic; 4: narrow ovate; 5: ovate; 6: broad ovate
Type of leaves	TL	1: even pinnate; 2: odd pinnate; 3: acacia-like leaflets
Growth habit	GH	1: bushy; 2: tall
*Reproductive phase*		
Flowering duration	FD	Record at the beginning to end of flowering (days)
Flower: color of standard and wings	FCS/ FCW	1: white; 2: creamy; 3: greenish; 4: pink; 5: reddish; 6: carmine; 7: light violet; 8: violet; 9: dark violet; 10: brown
Number of flowers in one inflorescence	NFI	1: one; 2: one or two; 3: two; 4: two or three; 5: three or more
Flower size	FS	1: small; 2: medium; 3: large
*Pod traits*		
Pod shattering	PShat	1: non-shattering; 2: shattering
Pod shape	PSh	1: straight; 2: slightly curved; 3: markedly curved
Pod length and width	PL, PW	The observations on well-developed green pods; the width is assessed from suture to suture on unopened pods.
*Seed traits*		
Ground color of testa	GCT	1: yellow; 2: green; 3: yellow and green; 4: yellow to greenish gray; 5: green-grey to brown-grey; 6: reddish brown-grey; 7: dark brown, brownish-yellow; 8: carmine
Pattern of the seeds	PS	0: absent; 1: present
Color of pattern of testa	CPT	0: absent; 1: violet; 2: brown; 3: violet and brown
Seeds shape	SSh	1: round, globular; 2: roundish-angular; 3: angular to compressed; 4: angular; 5: wrinkled; 6: granular surface
*Grain yield*		
Height to the first pod	HFP	Record at the height of first pod/ten plants per accession
Number of pods/plant	NPP	Record number of harvested pods/ten plants per accession
Number of grains/pod	NGP	Record number of seeds per pod/ten plants per accession
Number of grains/plant	NGPl	Record number of grains per plant/ten plants per accession
Weight of grains/plant	WGPl	Record weight of grains per plant//ten plants per accession
100-seed weight (g)	SW	Record the mass of 100 randomly taken seeds per accession
Harvest index (%)	HI	grain yield/total biomass × 100

**Table 4:** List of the twenty traits evaluated by correlation, cluster, PCA, and path analyses.

Trait	Abbreviation	Unit s
*Economic traits—Yield structure*		
Plant height	PlH	cm
Height to the first pod	HFP	cm
Number of branches	NB	
Number of pods per plant	NPPl	
Number of grains per pod	NGP	
Number of grains per plant	NGPl	
Weight of grains per plants	WGPl	g
Length of pod	PL	mm
Width of pod	PW	mm
100-seed weight	SW	g
Harvest index,	HI	%
*Biological traits—phenology*		
Germination–50% flowering	G-Fl	days
Duration of flowering	DFl	days
Growth cycle	GC	days
*Biochemical traits—seed components*		
Soluble protein	Pr	mg·g^−1^ FW
Soluble sugars	SS	mg·g^−1^ FW
Starch	St	mg·g^−1^ FW
Phenols	Phe	mg·g^−1^ FW
Free amino acids	AAs	mg·g^−1^ FW
Antitrypsin activity	AT	U·mg^−1^ FW

**Table 5:** Morphological evaluation of bitter vetch accessions—growth habit, stem, and leaves.

Accession	Anthocyanins	Growth Habit	Plant Branching	Leaf Color	Leaf Shape	Type of Leaf
A3BM0178	no	bushy	strongly from the base	green	narrow elliptic	even pinnate
BGR3051	yes	bushy	strongly from the base	green	narrow elliptic	odd pinnate
BGR3052	yes	bushy	strongly from the base	green	narrow elliptic	even pinnate
BGR6207	yes	bushy	strongly from the base	green	narrow elliptic	odd pinnate
BGR13526	no	bushy	strongly from the base	green	narrow elliptic	even pinnate
C3000001	no	tall	only in the upper part	green	elliptic	even pinnate
C3000002	no	bushy	strongly from the base	green	elliptic	even pinnate
C3000003	no	bushy	strongly from the base	yellow green	elliptic	even pinnate
C3000006	no	bushy	strongly from the base	yellow green	elliptic	odd pinnate
C3000007	no	bushy	strongly from the base	green	broad elliptic	odd pinnate
C3E0118	no	bushy	strongly from the base	yellow green	elliptic	even pinnate
B9E0168	no	bushy	strongly from the base	light green	narrow elliptic	even pinnate

**Table 6:** Morphological evaluation of bitter vetch accessions—flowers and grains.

Accession	Flower Size	Flowers in Inflorescence	Flowers: Color of Standard	Flowers: Color of Wings	Grain Shape	Ground Color of Testa	Testa Pattern
A3BM0178	medium	1–2	creamy	creamy	roundish-angular	reddish brown	no
BGR3051	medium	2–3	pink	pink	roundish-angular	reddish gray	no
BGR3052	medium	2–3	creamy brown streaks	creamy	roundish-angular	reddish gray	no
BGR6207	medium	1–2	creamy brown streaks	creamy	roundish-angular	reddish brown	no
BGR13526	small	1–2	pink	pink	round-globular	reddish brown	no
C3000001	medium	1–2	creamy brown streaks	creamy	round-globular	reddish brown	dots
C3000002	medium	2–3	light violet	creamy	round-globular	reddish gray	no
C3000003	medium	2–3	light violet	creamy	round-globular	reddish green brown	no
C3000006	medium	1–2	creamy brown streaks	creamy	round-globular	brownish gray	dots
C3000007	large	3 or more	light violet	creamy	round-globular	reddish gray	no
C3E0118	medium	2–3	creamy brown streaks	creamy	round-globular	reddish gray	no
B9E0168	medium	3 or more	creamy	creamy	roundish-angular	reddish brown	no

**Table 7:** Yield structure of the studied bitter vetch accessions, averaged for the years 2022 and 2023.

Accession	Plant Height, cm		Height to First pod, cm		Number of Branches		Number of Pods/Plant		Number of Grains/Pod		Number of Grains/Plant		Weight of Grains/Plant		Size of Pod				100-Seed Weight, g		Harvest Index, %	
															Length, mm		Width, mm					
	M	DM	M	DM	M	DM	M	DM	M	DM	M	DM	M	DM	M	DM	M	DM	M	DM	M	DM
A3BM0178	41.3		19.8		1.0		73.2		2.8		137.2		4.0		14.7		4.3		3.3		37.2	
BGR3051	50.7	9.4***	30.7	10.8***	1.2	0.2	32.8	−40.3***	2.1	−0.7***	55.0	−82.2***	1.1	−2.9***	12.5	−2.3***	3.9	−0.4***	2.2	−1.1***	11.3	−26.0***
BGR3052	50.0	8.7***	28.1	8.3***	3.6	2.6***	25.5	−47.7***	2.8	0.0	49.6	−87.6***	1.2	−2.9***	14.2	−0.5	3.9	−0.4***	2.6	−0.8***	19.8	−17.5***
BGR 6207	57.0	15.8***	24.0	4.2**	2.0	1.0**	105	31.3***	3.3	0.5***	226	89.2***	9.0	5.0***	18.8	4.1***	4.4	0.1	4.0	0.7***	41.1	3.9*
BGR13526	46.8	5.6**	24.6	4.8**	3.4	2.4***	41.4	−31.8***	2.8	0.0	63.3	−73.9**	2.3	−1.7	17.6	2.8***	4.5	0.2	3.8	0.5***	24.1	−13.1***
B9E0168	64.2	22.9***	30.8	11.0***	1.7	0.7*	97.5	24.3*	2.9	0.1	194	57.0**	6.2	2.1*	16.3	1.5**	4.2	−0.1	4.1	0.8***	26.4	−10.8***
C3000002	43.3	2.1	17.7	−2.2	1.3	0.3	65.3	−7.8	3.3	0.5***	180	42.8	6.2	2.2*	18.3	3.6***	4.5	0.2	3.9	0.6***	42.0	4.8*
C3000003	45.7	4.4*	20.0	0.2	4.0	3.0***	159	86.2***	4.0	1.2***	367	230.2***	13	9.4***	22.4	7.6***	5.0	0.7***	4.1	0.8***	31.8	−5.4**
C3000001	24.7	−16.6***	7.7	−12.2***	1.3	0.3	50.3	−22.8	3.2	0.4***	131	−6.2	6.7	2.7**	20.0	5.3***	4.5	0.2	4.4	1.0***	57.2	20.0***
C3000007	47.3	6.1**	27.3	7.5***	1.0	0.0	39.0	−34.2***	2.2	−0.6***	76.3	−60.8**	2.2	−1.8	13.1	−1.6***	3.8	−0.5***	3.1	−0.2	20.8	−16.4***
C3000006	28.3	−12.9***	10.3	−9.5***	0.7	−0.3	30.0	−43.2***	3.3	0.5***	83.7	−53.5**	4.3	0.3	18.5	3.7***	4.6	0.3**	4.6	1.2***	41.6	4.4**
C3E0118	48.3	7.0**	21.7	1.9	1.3	0.3	99.7	26.5*	3.3	0.5***	169	31.5	6.9	2.9***	16.2	1.5**	4.1	−0.2	3.5	0.2	38.1	0.9
average	45.6		21.9		1.9		68.2		3.0		144.4		5.3		16.9		4.3		3.6		32.6	
min	24.7		7.7		0.7		25.5		2.1		49.6		1.1		12.5		3.8		2.2		11.3	
max	64.2		30.8		4.0		159.3		4.0		367.3		13.4		22.4		5.0		4.6		57.2	
R	39.50		23.1		3.3		133.8		1.9		317.7		12.3		9.9		1.2		2.4		45.9	
CV, %	23.8		33.7		60.5		59.2		17.3		63.6		67.7		17.5		8.2		20.0		39.0	

Significant difference compared to the standard A3BM0178 at *p* = 0.1% *; *p* = 0.05% **; *p* = 0.01% ***; M—mean value; DM—difference regarding the mean value of the standard; CV—coefficient of variation; R—range of variation.

**Table 8:** Phenological data and grain biochemical composition of bitter vetch accessions, averaged for the years 2022 and 2023.

Accession	Germination to 50% Flowering, Days		Duration of Flowering, Days		Growth Cycle, Days		Soluble Protein, mg·g^−1^ FW		Sugars, mg·g^−1^ FW		Starch, mg·g^−1^ FW		Phenols, mg·g^−1^ FW		Amino Acids, mg·g^−1^ FW		TIA, Units mg^−1^ FW	
	M	MD	M	MD	M	MD	M	MD	M	MD	M	MD	M	MD	M	MD	M	MD
A3BM0178	73		14		112		105.4		28.73		571.13		0.219		0.030		1.967	
BGR3051	79	6	12	−2	116	4	100.8	4.60	35.55	−6.82**	561.75	9.38	0.210	−0.009	0.030	0.001	2.224	−0.590***
BGR3052	66	−7	8	−6	101	−11	86.05	19.35***	34.6	−5.87**	525.63	45.5	0.256	0.037	0.060	−0.022***	2.387	−1.293***
BGR 6207	80	7	11	3	108	−4	92.2	13.20***	33.55	−4.82	615.00	−43.87	0.260	0.041	0.040	−0.005	1.699	−0.502***
BGR13526	59	−14	6	−8	94	−18	86.05	19.35***	49.68	−20.95***	627.75	−56.62	0.329	0.110***	0.040	−0.011	1.22	0.502***
B9E0168	83	10	11	−3	162	3	105.8	−0.40	35.43	−6.70**	565.50	5.63	0.206	−0.014	0.050	−0.001	2.178	−0.053
C3000002	54	−29	8	−6	84	−28	95.9	9.50***	38.40	−9.67***	506.73	64.4**	0.197	−0.022	0.060	−0.027***	2.550	−0.308***
C3000003	67	−6	13	−1	83	−29	88.1	17.30***	36.20	−7.47***	591.77	−20.64	0.145	−0.074***	0.077	−0.044***	2.370	−0.133
C3000001	41	−32	10	−4	83	−29	92.7	12.70***	26.07	2.66	598.90	−27.77	0.180	−0.040	0.068	−0.035***	2.390	−0.153
C3000007	67	−6	8	−6	83	−29	85.7	19.70***	41.33	−12.6***	502.63	68.5**	0.142	−0.078***	0.056	−0.023***	2.140	0.100
C3000006	41	−32	12	−2	83	−29	93.85	11.55***	21.67	7.06**	581.70	−10.57	0.211	−0.009	0.061	−0.0280***	2.080	0.158**
C3E0118	162	121	14	0	210	98	89.2	16.20***	29.87	−1.14	509.73	61.4**	0.147	−0.072***	0.072	−0.039***	2.500	−0.263***
average	64.5		10.3		97.5		93.48		34.26		563.19		0.209		0.054		2.142	
min	41		6		83		85.70		21.67		502.63		0.142		0.030		1.220	
max	83		14		162		105.8		49.68		627.75		0.329		0.077		2.550	
R	42		8		33		20.10		28.01		125.12		0.187		0.047		1.330	
CV, %	22.6		24.3		14.7		6.8		21.4		7.7		26.3		29.4		17.6	

Significant difference compared to the standard A3BM0178 at *p* = 0.05% **; *p* = 0.01% ***; M—mean value; DM—difference regarding the mean value of the standard; CV—coefficient of variation; R—range of variation.

**Table 9:** Phenotypic correlation of different characters in bitter vetch.

	PlH	HFP	NB	NPPl	NGP	NGPl	WGPl	PL	PW	SW	HI	G-Fl	DFl	GC	Pr	SS	St	Phe	AAs	AT
PlH	1	0.898**	0.286	0.337	−0.222	0.195	0.017	−0.333	−0.372	−0.371	0.602*	0.466	−0.042	0.319	0.152	0.509	−0.132	0.189	−0.328	−0.163
HFP		1	0.271	0.015	−0.554	−0.136	−0.332	−0.627*	−0.613*	−0.682*	−0.869**	0.370	−0.166	0.237	0.134	0.600*	−0.233	0.214	−0.444	−0.181
NB			1	0.321	0.338	0.312	0.256	0.342	0.316	−0.055	−0.298	−0.077	−0.301	−0.171	−0.368	0.496	0.312	0.360	0.214	−0.206
NPPl				1	0.695*	0.963**	0.910**	0.583*	0.538	0.369	0.233	0.380	0.513	0.239	−0.074	−0.014	0.178	−0.363	0.327	0.145
NGP					1	0.782**	0.867**	0.907**	0.839**	0.719**	0.640*	0.021	0.308	0.007	−0.241	−0.304	0.269	−0.191	0.639*	0.209
NGPl						1	0.965**	0.711**	0.661*	0.474	0.342	0.166	0.435	0.028	−0.089	−0.067	0.191	−0.394	0.412	0.225
WGPl							1	0.828**	0.734**	0.617*	0.521	0.132	0.431	0.032	−0.171	−0.194	0.273	−0.398	0.517	0.219
PL								1	0.933**	0.828**	0.664*	−0.256	0.096	−0.266	−0.181	−0.163	0.514	−0.080	0.544	0.009
PW									1	0.771**	0.560	−0.346	0.165	−0.343	−0.029	−0.13	0.581*	0.009	0.349	−0.112
SW										1	0.758**	−0.275	0.066	−0.236	0.033	−0.295	0.444	−0.064	0.426	−0.105
HI											1	0.140	0.220	−0.036	−0.11	−0.576	0.222	−0.200	0.396	0.178
G-Fl												1	0.472	0.964**	−0.07	−0.025	−0.369	−0.280	0.099	0.198
DFl													1	0.496	0.193	−0.671*	0.035	−0.486	0.052	0.318
GC														1	0.022	−0.167	−0.313	−0.190	0.061	0.19
Pr															1	−0.088	0.081	0.070	−0.397	0.046
SS																1	0.002	0.367	−0.241	−0.443
St																	1	0.530	−0.288	−0.677*
Phe																		1	−0.584*	−0.756**
AAs																			1	0.611*
AT																				1

** Correlation is significant at the 0.01 level (2-tailed); * Correlation is significant at the 0.05 level (2-tailed). Significant correlations are in bold. Abbreviations: PlH—plant height, cm; HFP—height to the first pod, cm; NB—number of branches; NPPl—number of pods per plant; NGP—number of grains per pod; NGPl—number of grains per plant; WGPl—weight of grains per plants, g; PL—size of pod, length, mm; PW—size of pod, width, mm; SW—weight of 100 seeds, g; HI—harvest index, %; G-Fl—germination−50% flowering, days; DFl—duration of flowering, days; GC—growth cycle, days; Pr—protein, mg g^−1^ FW; SS—soluble sugars, mg·g^−1^ FW; St—starch, mg·g^−1^ FW; Phe—phenols, mg·g^−1^ FW; AAs—amino acids, mg·g^−1^ FW; AT—antitrypsin activity, U·mg^−1^ FW.

**Table 10:** Distribution of the total variation among components.

Component	Total	Initial Eigenvalues	
		% of Variance	Cumulative %
PC1	7.259	**36.294**	36.294
PC2	3.98	**19.899**	56.193
PC3	3.375	**16.875**	73.068

The contribution of each component in the total variation is marked in bold.

**Table 11:** Factor loadings derived from PCA performed on 12 bitter vetch accessions based on 20 agro-morphological and biochemical traits.

Traits/Components	PC1	PC2	PC3
Plant height, cm	−0.398	0.300	**0.773**
Height to the first pod, cm	**−0.706**	0.232	0.633
Number of branches	0.129	−0.323	**0.721**
Number of pods per plant	**0.662**	0.372	0.609
Number of grains per pod	**0.936**	0.021	0.191
Number of grains per plant	**0.774**	0.258	0.500
Weight of grains per plants, g	**0.888**	0.197	0.364
length of pod, mm	**0.933**	−0.284	0.158
width of pod, mm	**0.867**	−0.370	0.152
Weight of 100 grains, g	**0.812**	−0.290	−0.107
Harvest index, %	**0.765**	−0.065	−0.435
Germination−50% flowering, days	−0.103	**0.794**	0.316
Duration of flowering, days	0.374	**0.642**	−0.068
Growth cycle, days	−0.107	**0.742**	0.146
Protein,mg·g^−1^ FW %	−0.056	**0.233**	−0.124
Soluble sugars, mg·g^−1^ FW	−0.419	−0.328	**0.641**
Starch, mg·g^−1^ FW	0.334	**−0.643**	0.25
Phenols, mg·g^−1^ FW	−0.348	**−0.701**	0.268
Amino acids, mg·g^−1^ FW	**0.623**	0.265	−0.106
Antitrypsin activity, U·mg^−1^ FW	0.233	**0.673**	−0.347
Extraction Method: Principal Component Analysis.			
a. 3 components extracted.			

The bolded values determine which factor the traits fall into.

**Table 12:** Direct and indirect effects of economic traits on grain yield per plant in bitter vetch.

	Direct and Indirect Effect								Total Indirect Effect	Total Phenotypic Correlation with WGPl
	Plant Height	Height to First Pod	Number of Main Branches	Number of Pods/Plant	Number of Grains per Pod	Number of Grains per Plant	100-Seed Weight	Harvest Index		
**PlH**	**−0.324**	−0.291	−0.093	−0.109	0.072	−0.063	0.120	0.195	−0.169	0.017
**HFP**	0.384	**0.428**	0.116	0.006	−0.237	−0.058	−0.292	−0.372	−0.452	−0.332
**NB**	−0.003	−0.003	**−0.010**	−0.003	−0.003	−0.003	0.001	0.003	−0.012	0.256
**NPPl**	0.036	0.002	0.034	**0.106**	0.073	0.102	0.039	0.025	0.310	0.910**
**NGP**	−0.038	−0.095	0.058	0.119	**0.172**	0.134	0.123	0.110	0.412	0.867**
**NGPl**	0.141	−0.099	0.226	**0.698**	**0.566**	**0.724**	0.521	0.248	2.301	0.965**
**SW**	−0.039	−0.071	−0.006	0.039	0.075	0.050	**0.105**	0.079	0.127	0.617*
**HI**	−0.140	−0.203	−0.069	0.054	0.149	0.080	0.177	**0.233**	0.048	0.521

** Correlation coefficient is significant at the 0.01 level (2-tailed); * correlation is significant at the 0.05 level (1-tailed); underlined and bolded values are direct effects and all other values are indirect effects of economic traits on grain yield per plant. Abbreviations: PlH—plant height; HFP—height to the first pod; NB—number of branches; NPPl—number of pods per plant; NGP—number of grains per pod; NGPl—number of grains per plant; SW—weight of 100 seeds, g; HI—harvest index, WGPl—weight of the grains per plant.

**Table 13:** Direct and indirect effects of biological and biochemical traits on grain yield per plant in bitter vetch.

		Direct and Indirect Effect								Total Indirect Effect	Total Phenotypic Correlation with WGPl
	Germination- 50%fl.	Flower duration	Growth Cycle	Protein	Sugars	Starch	Phenols	Amino Acids	Anti- trypsins		
G-Fl	**2.262**	1.068	2.181	−0.025	−0.057	−0.835	−0.633	0.224	0.448	2.371	0.132
DFl	0.128	**0.271**	0.134	0.099	−0.182	0.009	−0.132	0.014	0.086	0.157	0.431
GC	−2.074	−1.067	**−2.152**	−0.123	0.359	**0.673**	0.409	−0.131	−0.409	−2.362	0.032
Pr	−0.002	0.082	0.013	**0.224**	−0.064	0.012	−0.010	−0.089	0.036	−0.022	−0.005
SS	0.002	0.053	0.013	0.023	**−0.079**	0.000	−0.029	0.019	0.035	0.116	−0.194
St	−0.233	0.022	−0.198	0.035	0.001	**0.631**	0.334	−0.182	−0.427	−0.647	0.273
Phe	−0.053	−0.093	−0.036	−0.008	0.070	0.101	**0.191**	−0.112	−0.144	−0.275	−0.398
AAs	0.065	0.034	0.040	−0.260	−0.158	−0.189	−0.383	**0.655**	0.400	−0.450	0.517
AT	0.038	0.061	0.037	0.031	−0.086	−0.131	−0.146	0.118	**0.193**	−0.077	**0.219**

Underlined and bolded values are direct effects and all other values are indirect effects of biological and biochemical traits on grain yield per plant. Abbreviations: G-Fl—germination−50% flowering; DFl—duration of flowering; GC—growth cycle; Pr—seed soluble protein; SS—seed soluble sugars; St—starch; Phe—Phenols; AAs—free amino acids; AT—antitrypsin activity, WGPl—weight of the grains/plant.

**Table 14:** Germination percentage, vigor index (VI), and relative stress tolerance (RST) among the twelve studied bitter vetch accessions.

Accession	Germination %			RST-G %		Seedling VI			RST-VI %	
	H_2_Od	PEG	NaCl	PEG	NaCl	H_2_Od	PEG	NaCl	PEG	NaCl
A3BM0178	83.17	86.91	55.84	1.045	0.671	6.82	6.16	0.95***	0.903	0.139
BGR3051	42.24	29.60*	30.47	0.701	0.721	2.88	1.66	1.20***	0.576	0.417
BGR3052	93.94	91.16	82.58	0.970	0.879	6.85	4.53**	1.83***	0.661	0.267
BGR 6207	71.86	67.59	44.38	0.941	0.617	5.28	3.64*	1.36***	0.689	0.258
BGR13526	70.91	72.40	32.07**	1.021	0.452	5.22	6.18	2.28***	1.184	0.437
C3000001	79.92	61.39	15.00**	0.768	0.188	5.44	4.01	0.11***	0.737	0.020
C3000002	95.00	98.04	63.49*	1.032	0.668	8.26	5.47***	1.54***	0.662	0.186
C3000003	95.96	73.44	69.34*	0.765	0.723	7.52	4.30*	2.23***	0.572	0.297
C3000006	56.15	66.57	5.83**	1.186	0.104	3.13	3.54	nd	1.131	nd
C3000007	80.58	53.70*	0.00***	0.666	0.000	7.51	3.09**	nd	0.411	nd
C3E0118	93.65	80.32	52.38*	0.857	0.559	7.29	4.77*	1.62***	0.743	0.222
B9E0168	88.47	79.33	50.84*	0.897	0.575	6.03	4.48***	1.17***	0.744	0.194
average	79.32	71.70	41.85	0.904	0.513	6.02	4.32	1.19	0.751	0.203
CV%	15.98	18.51	43.68	14.22	42.75	22.84	21.88	62.06	22.04	55.66

Values are mean from 3 independent experiments. Significant differences (one way ANOVA) compared to control (distilled water) for each accession are marked with asterisk (at *p* = 0.1% *; *p* = 0.05% **; *p* = 0.01% ***), nd—not determined.

**Table 15:** Growth parameters of the germinated bitter vetch plants.

Accession	Root Length (mm)			Shoot Length (mm)			Root/Shoot Ratio		
	H_2_Od	PEG	NaCl	H_2_Od	PEG	NaCl	H_2_Od	PEG	NaCl
A3BM0178	48	59***	15***	37	30	6***	1. 511	2.266***	2.778***
BGR3051	43	72**	17**	50	54	21**	0.903	1.401*	0.905
BGR3052	49	53	12***	49	46	10***	1.080	1.279	1.341
BGR 6207	36	41	14***	35	44	14***	1.032	1.018	1.235
BGR13526	25	66**	24	27	46*	36	1.104	1.552	0.703
C3000001	35	55**	nd	29	26	nd	1.256	2.410***	nd
C3000002	49	43	9***	33	38	6***	1.649	1.232*	1.303
C3000003	45	80***	20***	40	41	12***	1.255	2.233***	2.207*
C3000006	40	53	22	24	25	15	1.791	2.267	1.470
C3000007	53	53	nd	37	24*	nd	1.485	2.091*	nd
C3E0118	28	40**	10***	30	23	14**	1.084	2.045**	0.847
B9E0168	43	53	10***	42	44	10***	1.103	1.248	1.200
average	41	56	15	36	37	14	1.246	1.753	1.399
CV%	17	16	28	18	25	40	17.57	26.52	32.36

Values are the means from independent measurements of all germinated plants that had developed roots and shoots upon the respective treatment in one representative experiment; nd—not determined. Significant differences (one-way ANOVA) compared to control (distilled water) for each accession are marked with asterisk (at *p* = 0.05% *; *p* = 0.01% **; *p* = 0.001% ***).

## Data Availability

The original contributions presented in this study are included in the article/Supplementary Materials.

## Supplementary Materials

The following supporting information can be downloaded at https://www.mdpi.com/article/10.3390/plants14071008/s1. Figure S1. Phenotypic variation in the studied parameters; Figure S2. Projection of the studied features in the PCA factor plane; Table S1. Weighted factors (PC1, PC2, and PC3) of the studied accessions; Figure S3. Germination in dependence on the stress intensity in bitter vetch variety Rodopi; Figure S4. Bitter vetch germination dynamics in control conditions; Figure S5. Bitter vetch G % in the studied accessions under osmotic stress (0, 10, 15, and 20% PEG 6000); Figure S6. Bitter vetch G % in the studied accessions under salt stress (0, 75, and 150 mM NaCl); Figure S7. Bitter vetch vigor index under osmotic stress (0, 10, 15, and 20% PEG 6000); Figure S8. Bitter vetch vigor index in the studied accessions under salt stress (0, 75, and 150 mM NaCl).
