# Supplementary material for: Phenotypic Diversity and Abiotic Stress Tolerance Among Vicia ervilia (L.) Willd. Accessions
Source: Plants (Basel). 2025 Mar 24;14(7):1008. doi: 10.3390/plants14071008 (PMC11990473; doi:10.3390/plants14071008)
Supplement: Supplementary file 1 [file plants-14-01008-s001.zip › plants-3504791-supplementary.pdf]

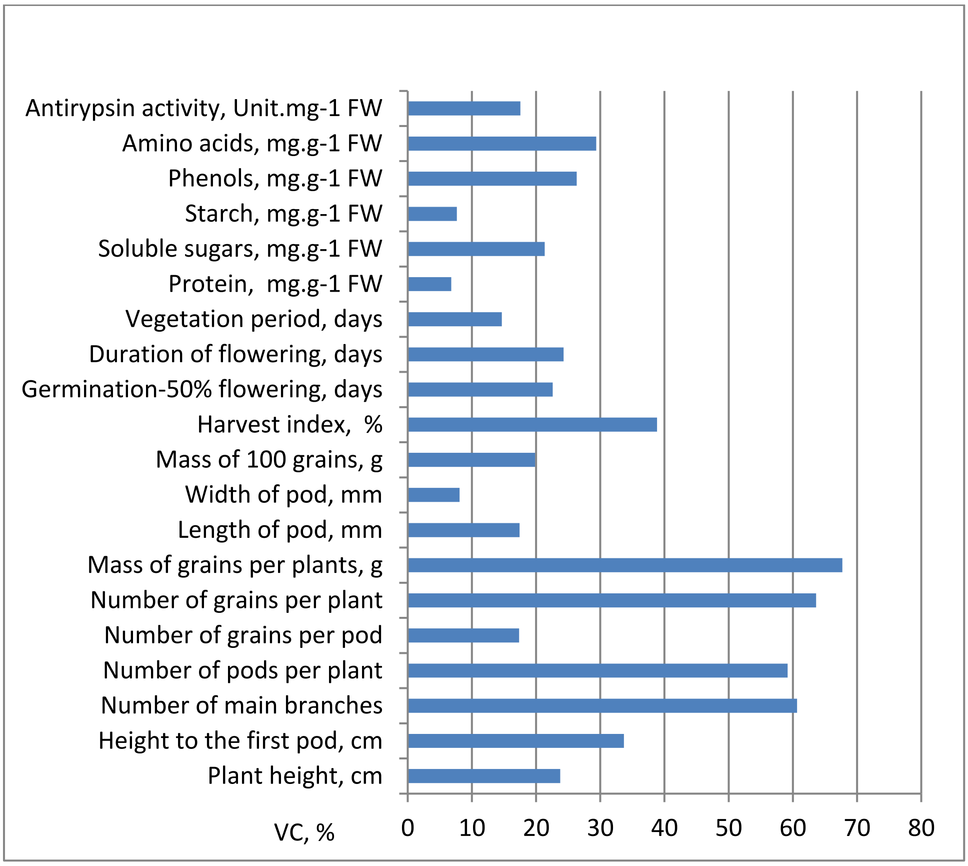

Figure S1. Phenotypic variation of the studied characters

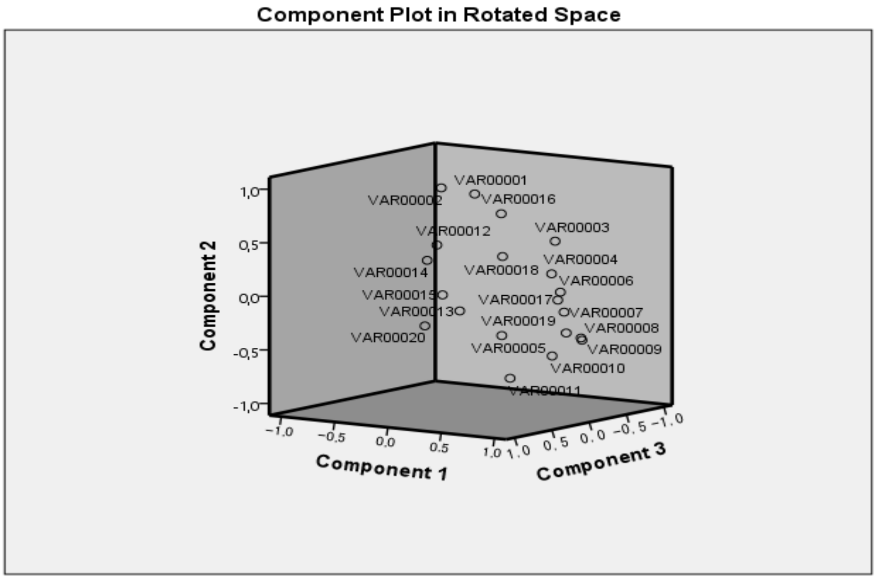

Figure S2. Projection of the studied features in the factor plane . **Var 00001** Plant height, cm; **Var 00002** Height to the first pod, cm; **Var 00003** Number of branches; **Var 00004** Number of pods per plant; **Var 00005** Number of grains per pod; **Var 00006** Number of grains per plant; **Var 00007** Mass of grains per plants, g; **Var 00008** Size of pod- length, mm; **Var 00009** Size of pod- width, mm; **Var 000010** Mass of 100 grains, g; **Var 000011** Harvest index, %; **Var 000012** Germination-50% flowering, days; **Var 000013** Duration of flowering, days; **Var 000014** Vegetation

period, days; **Var 000015** Protein, mg g^{-1} FW; **Var 000016** Soluble sugars, mg g^{-1} FW; **Var 000017** Starch, mg g^{-1} FW; **Var 000018** Phenols, mg g^{-1} FW; **Var 000019** Amino acids, mg g^{-1} FW; **Var 000020** Antitrypsin activity, U mg^{-1} FW

Table S1. Weighted factors (PC1, PC2 and PC3) of the studied accessions with three factors

Accessions/Components	PC1	PC2	PC3
1-A3BM0178	**-0.398**	-0.245	0.309
2-BGR3051	**-1.370**	0.799	0.432
3-BGR3052	**-0.876**	0.590	-0.250
4-BGR 6207	**0.843**	0.509	-0.622
5-BGR13526	-0.230	0.636	**-2.360**
6-B9E0168	0.311	**0.927**	0.282
7-C3000002	0.130	**-0.562**	0.106
8-C3000003	**2.374**	0.368	-0.158
9-C3000001	-0.131	**-2.000**	-0.052
10-C3000007	**-1.165**	0.299	0.275
11-C3000006	-0.249	**-1.889**	-0.083
12-C3E0118	0.498	0.569	**2.121**

The most important contributions to each PC are marked with bold

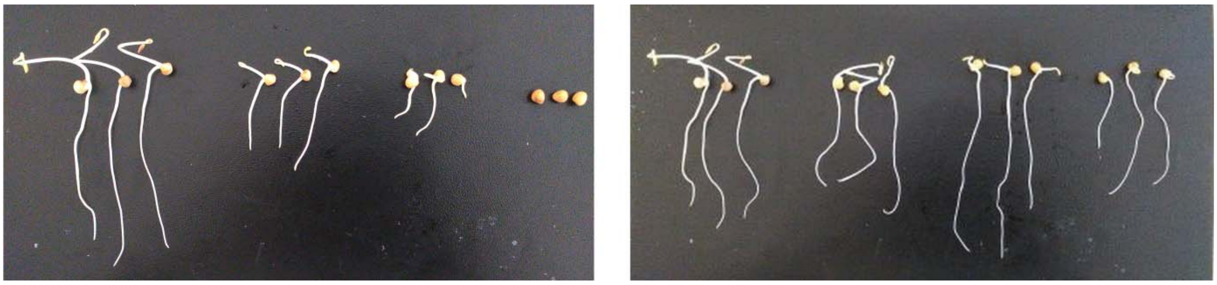

Figure S3. Germination (120 h) in dependence of the stress intensity in bitter vetch variety Rodopi.
Left part – NaCl stress, from left to right: controls, 75 mM, 150 mM, and 200 mM NaCl.
Right part – PEG 6000 w/v, from left to right: controls, 5%, 10%, 15% PEG.

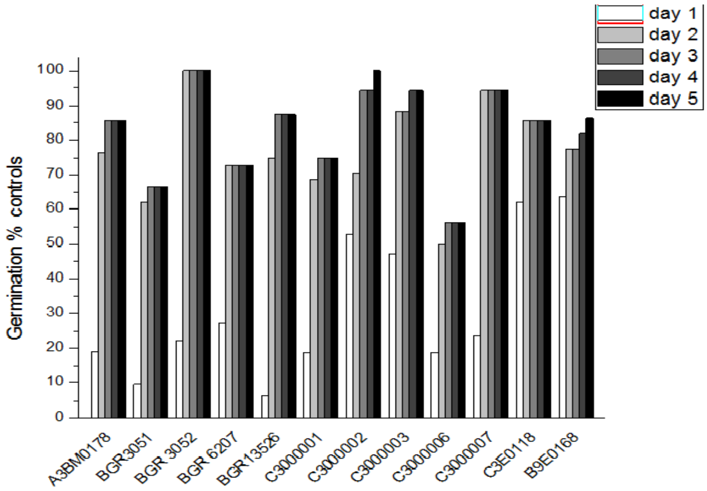

Figure S4. Bitter vetch germination dynamics in control conditions. X axis – accessions, Y axis – germination %. White columns - first 24 h, daily dynamics is marked with different shades of gray from lightest to darkest

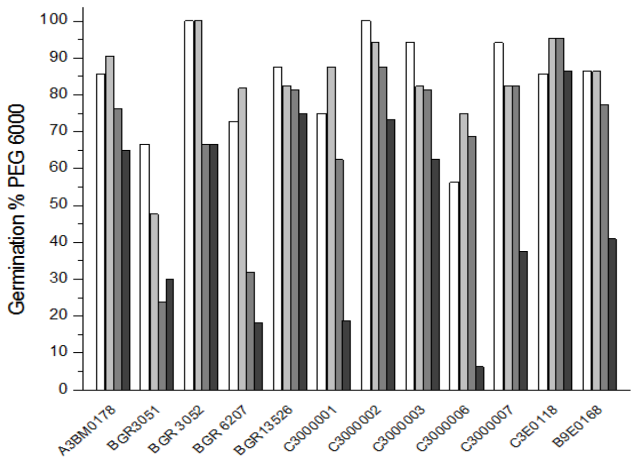

Figure S5. Bitter vetch germination % (120 h), white columns – H_2Odist., light gray – 10% PEG, gray – 15% PEG, dark gray – 20% PEG. . X axis – accessions

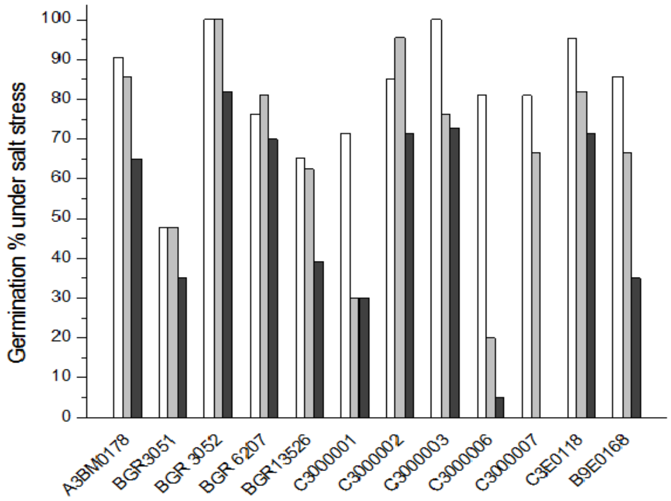

Figure S6. Bitter vetch germination % (120 h), under salt stress. White columns – H_2Od, light gray – 75 mM NaCl, gray – 150 mM NaCl. X axis – accessions

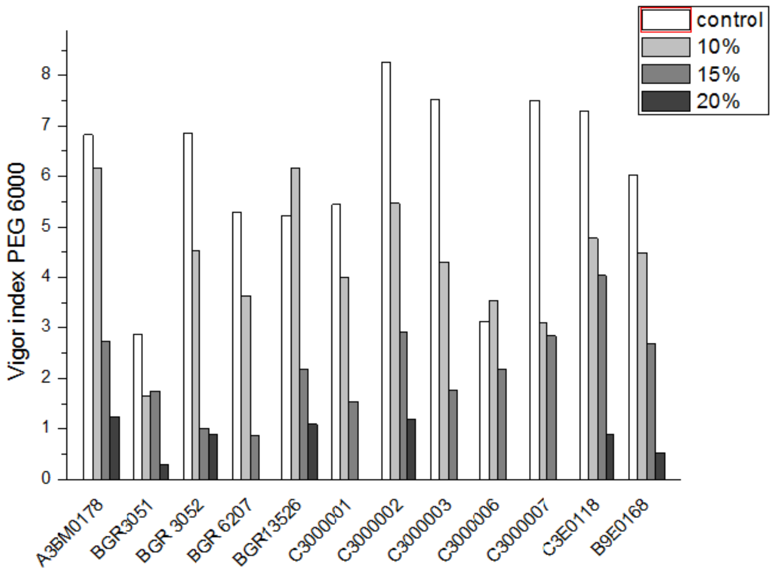

Figure S7. Bitter vetch vigor index under osmotic stress (120 h). White columns – – H_2Od, light gray – 10% PEG, gray – 15% PEG, dark gray – 20% PEG. . X axis – accessions

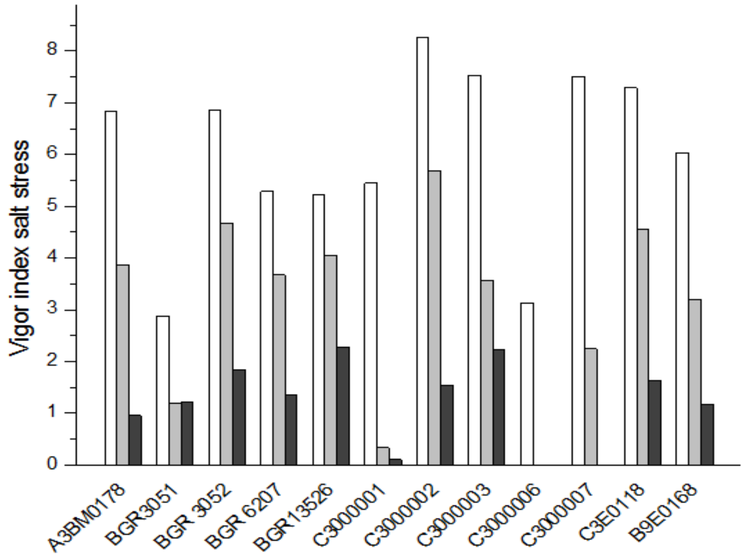

Figure S8. Vigor index under salt stress. White columns – H_2Od, light gray – 75 mM NaCl, gray – 150 mM NaCl. . X axis – accessions
